# Effects of the COVID-19 Pandemic on Dairy Consumption Trends: An Empirical Investigation of Accounting Data in China

**DOI:** 10.3390/foods13050741

**Published:** 2024-02-28

**Authors:** Jianxiong Chen, Chung-Cheng Yang, Yu Lin

**Affiliations:** 1 College of Economics and Management, Fujian Agriculture and Forestry University, Fuzhou 350002, China; jianxiong@fafu.edu.cn; 2 College of Management, National Yunlin University of Science and Technology, Douliu 64002, Taiwan; pfc@gapp.nthu.edu.tw

**Keywords:** consumption trend, COVID-19, the global pandemic, dairy products, eating behavior

## Abstract

The COVID-19 pandemic may have had a negative impact on dairy consumption trends. Many dairy products are perishable and have relatively high income elasticity, causing their susceptibility to market fluctuations in general, including those specifically caused by the pandemic. However, the pandemic has also brought some other prospective possibilities. For example, during the pandemic, people paid more attention to nutrition and health issues and increased the number of meals prepared and eaten at home. In consideration of the particular circumstances during the pandemic, the Chinese government issued several policies to promote the population’s dairy consumption, and the Chinese dairy cattle sector actively implemented the policy of “guarantee price, quality, and supply”. These factors may have caused the Chinese population to increase their consumption of dairy products during the pandemic. Before the pandemic, the consumption of dairy products in the Chinese population showed an overall upward trend. The question addressed in this study is how has COVID-19 affected dairy consumption trends during the pandemic? This study uses accounting data from the Chinese dairy cattle sector to empirically analyze the impact of the COVID-19 pandemic on dairy consumption trends through economic theories and translog revenue function. Our study found that COVID-19 increased consumers’ consumption of dairy products in China, but those people experiencing poverty may still have experienced inadequate dairy intake. This study has contributed to the body of work in this area in the literature and provides response strategies for the dairy cattle sector and the authorities.

## 1. Introduction

During the COVID-19 pandemic and the implementation of restrictive measures to control its spread, consumers’ preparation and intake of food were often impacted. Implementing business closures, social distancing, and border closures had the effect of significantly reducing economic activity. This, in turn, affected people’s household income and spending, including their food consumption. Despite its basic necessity, food consumption was not immune to the global crisis induced by COVID-19. In China, a range of factors, including reduced incomes due to the inability to work [1,2], increased expenditure on medical care [3], and fear [4], led to a decline in food consumption during the pandemic. Consumers were observed making changes to their food consumption preferences, opting for more cost-effective alternatives [5] that included a reduction in fruit consumption [6] and a decrease in the intake of animal-derived foods [6,7]. They also became more reliant on shelf-stable packaged foods [8]. The COVID-19 pandemic further altered consumer dietary patterns and food-sourcing strategies, and consumers may now prefer to procure their food through online channels that offer convenient door-to-door delivery services, which are perceived as safer [9] and more comfortable [10]; therefore, online food deliveries have become more desirable. Moreover, consumers may now choose the channels that allow them to maintain higher household inventories of staple goods to minimize their need for frequent visits to physical markets.

These changes may be more visible and prominent in the household consumption of dairy products. Dairy products have both higher income elasticity and price elasticity. As per a research survey, the household consumption of dairy products decreased by 11 percentage points from the onset of the COVID-19 pandemic. This decrease can be attributed to the perceived risk of viral infection while visiting stores in person to purchase food [7]. A study also reported an overall decline in the consumption of dairy products in some Latin American countries [11]. In China, consumption trends of animal-derived foods were severely impacted during and as a result of both the SARS and COVID-19 outbreaks. During the SARS pandemic, concerns over the airborne transmission of the virus led to a reduction in social interactions and outdoor activities. Furthermore, the uncertain economic outlook resulted in lowered income expectations, which in turn caused a significant decline in consumer spending [12], and people’s consumption of many foods decreased [13]. However, the Chinese population increased their consumption of dairy products at the same time, which also prompted the sales revenue growth rate of Chinese dairy companies to reach its highest level at that time [14]. Yili, a leading Chinese dairy company, showed high sales performance during the SARS pandemic [15]. In addition, during the SARS pandemic, Sanyuan Company, another major dairy production enterprise in China, also stated that the sales of dairy products had increased by 20% [16].

Nearly 20 years after the occurrence of SARS, the Chinese market and per capita consumption levels have further improved. At the time of the COVID-19 outbreak in 2020, the per capita disposable income of Chinese residents was RMB 32,189, which is 3.9 times higher than during the SARS pandemic after adjusting for inflation. With the increase in income levels, Chinese residents now pay more attention to their health, and the consumption of dairy products has also increased [14]. Before the pandemic, the consumption of dairy products in the Chinese population showed an overall upward trend, and dairy products had become one of the sources of protein for an increasing number of people [14]. During the COVID-19 pandemic, taking into account the particular circumstances of the pandemic, the Chinese government issued a series of national policies to guarantee and promote dairy consumption. For example, for the first time, milk was designated as a necessity by the State Council of China [17], and was recommended by the Chinese Center for Disease Control and Prevention as an important food for residents to consume in relation to the prevention and control of pandemics. It was also listed as one of the key guaranteed foods for pandemic prevention and control by the China Development and Reform Commission [18], and these national policies all had a positive significance in the promotion of dairy consumption [14].

At present, scholars have completed limited research on the changes in dairy consumption during the COVID-19 pandemic. However, with increasing numbers of consumers recognizing the nutritional benefits provided by the consumption of dairy products [19,20], it is of great significance to explore the impact of COVID-19 on the trend of dairy consumption. The objective of our research is to investigate the impact of the COVID-19 pandemic on the consumption trends in dairy products. The results of this study contribute to the literature and provide policy recommendations for dairy companies and authorities.

The following sections of this paper will be structured in the following manner. Section 2 introduces the background and develops our hypothesis, providing a comprehensive overview. Section 3 encompasses the variable definitions, data sources, and the specific model derivation process, ensuring a rigorous methodology. Section 4 showcases the findings of this study, presenting insightful analysis. The present study concludes by elucidating the deep-rooted influence of the COVID-19 pandemic on dairy consumption while putting forward substantive suggestions for future research.

## 2. Background and Hypothesis Development

The National Dairy Industry Development Plan 2016–2020 [21] pointed out that in recent years, China’s dairy industry has made significant progress in modernization, and the supply-side structural reform of the dairy industry has achieved substantial results, with supply and consumer demand becoming more compatible. In terms of industrial structure, competition in China’s dairy industry is fierce. The current Chinese dairy industry presents a competitive landscape in which large state-level companies compete with local, small, and medium-sized enterprises. The Ministry of Industry and Information Technology of the People’s Republic of China [22] pointed out that in 2016, the industry concentration of China’s top 10 dairy companies exceeded 65%, and the market concentration of high-end milk was very high [23]. According to statistics of the market sales of large-scale retail enterprises in China, it was found that the top 10 Chinese dairy product sales companies (9 of which are listed companies) have a combined share of 90.01% in the high-end milk market [22]. China Business Radio [24] reported that Yili, the largest listed dairy product company in China, has a market share of approximately 70% in China’s organic milk market. In 2017, the industry concentration of China’s top 10 dairy companies remained at around 65% [25]. In 2019, the total market share of two listed dairy companies, Yili and Mengniu, accounted for nearly 50% [26]. It can be seen that the overall production and sales of dairy products of the listed Chinese dairy products companies are highly representative of the industry as a whole.

In terms of business structure, taking Yili and Mengniu as examples, their products mainly include liquid milk, milk beverages, yogurts, and milk powder. The Analysis Report on the Market Competition and Investment Strategy of China’s Liquid Milk Industry from 2021 to 2027 [27] shows that liquid milk is the main source of revenue for Yili and Mengniu. In 2020, liquid milk accounted for 78.9% of Yili’s total revenue, and liquid milk accounted for 89.1% of Mengniu’s total revenue. The above ratios are only the share of liquid milk and do not include the share of other dairy products. Thus, it can be seen that dairy products account for most of the company’s sales revenue.

Dairy products have an irreplaceable position in the nutritional intake of the population [28,29,30,31,32,33]. Dairy products are necessary for healthy development and growth, especially for children [34,35,36]. The nutritional value of dairy products and their rich vitamin and mineral content [37,38,39,40,41] make a significant contribution to consumer health [42,43]. Many components in dairy products have been shown to have significant health promotion effects, such as milk protein [44,45,46,47,48], milk fat [40,49,50], carbohydrates [51], minerals [31,49,52,53,54], and vitamins [49,52,55,56,57,58]. These components of dairy products play a very positive role in helping to reduce malnourishment worldwide [59]. A large number of epidemiological studies have shown that the consumption of dairy products can reduce systolic blood pressure [60,61,62], the risk of type 2 diabetes [63,64,65], colorectal cancer [66], stroke, and heart disease [64,65]. Based on these proven positive nutritional effects of dairy products, it can be said that the consumption of dairy products has brought beneficial health effects to consumers of all ages, especially children. The National Promotion Plan of “Student Drinking Milk Plan” (2021–2025) proposes that more than 60 countries promote student drinking milk, benefiting more than 160 million children, a critical way to improve and improve students’ nutritional health and physical fitness. China’s child malnutrition has yet to be fundamentally solved, and the role of students drinking milk in improving nutrition is crucial.

Many scholars have discussed the relevant factors affecting the consumption of dairy products. Alwis et al. [67] reported that the price level and availability of dairy products are the main factors affecting people’s purchase and consumption of dairy products. Grebitus et al. [68] pointed out that price, freshness, and shelf life are the most important factors affecting the purchasing decision of dairy products, and the least important determinant is additional information, such as recipes. Boniface et al. [69] emphasized that for consumers, the price information of dairy products has a direct impact on purchasing decisions. Špička [70] wrote that as financial conditions deteriorate, consumers will buy cheaper private-label dairy products. According to the research of Kurajdová et al. [71], consumers attach great importance to the health and taste of dairy products, and these two factors directly affect consumers’ purchasing decisions. Matulová et al. [72] reported that the price of dairy products is affected by the existence of retail chains, and the number of retail chains is closely related to the price of dairy products. Retail chain stores can transfer the effects of market changes (mainly negative effects) to dairy product companies. Based on the market structure for dairy products, dairy product companies further transfer these negative price changes to farmers. The above-mentioned publications have discussed the factors affecting the consumption of dairy products from many perspectives.

The emergence of the new coronavirus rapidly became a global health problem [73,74] and the World Health Organization declared the COVID-19 pandemic to be a significant public health emergency [75]. This outbreak significantly impacted agriculture [76,77,78,79,80,81,82,83,84], particularly in the dairy industry, which was one of the worst affected. The supply chain of the dairy industry is time-sensitive, and many dairy products can spoil quickly [85,86,87,88,89,90,91,92]. Due to the COVID-19 pandemic, many countries have experienced a notable increase in unemployment rates, significantly impacting the global economy. The International Labour Organization [93] reported that working hours were down 10.4% in 2020 compared to its previously published findings. As a result, the global pandemic has hindered numerous individuals’ participation in the workforce, which may lead to a decline in consumers’ purchasing power of dairy products.

The COVID-19 pandemic significantly impacted consumer behavior, particularly with regard to the consumption of dairy products. With personal safety being a top priority, people reduced their visits to physical markets to purchase dairy products. Additionally, the pandemic’s adverse effects on global income and future income uncertainty led to a conscious reduction in expenditures on animal-derived foods. People increasingly attempted to save money to cope with the economic risks associated with the pandemic, which may have further contributed to the decline in dairy consumption. Studies conducted in countries such as Ethiopia [7] and Guatemala [11] have reported a negative impact on the dairy product consumer market due to the COVID-19 pandemic. Despite the above findings, all the findings that seem to have negative impacts on the dairy industry were not evident in China; the Chinese dairy produce market during the pandemic had a completely different sales situation from many other countries.

There are multiple reasons for the above differences. Firstly, the Chinese economy’s robust driving force and the high savings rate among Chinese citizens contributed to a swift recovery in consumer willingness to spend. This, in turn, positively impacted dairy product sales in the country. As per a survey conducted in China regarding consumer attitudes [94], it was observed that consumer confidence gradually recovered as the negative impacts of the pandemic waned. The survey involved approximately 2500 Chinese consumers who were asked about their use of consumer products. Based on the feedback received, the majority of respondents anticipated that the usage and consumption of consumer products would revert to the levels observed before the pandemic. Approximately 60% to 70% of respondents expected that the consumption of consumer products would either return to normal levels or slightly increase. Furthermore, 10% of the respondents indicated that consumption of consumer products would significantly increase, reflecting a potential delay in demand. Only 20% to 30% of respondents reported continuing to be cautious and slightly or substantially reducing their consumption of various consumer products. On the other hand, Chinese companies responded to the potential adverse effects of the pandemic by taking positive measures, such as increasing their marketing and promotion efforts to boost consumption.

Second, from the perspective of the supply side of China’s dairy product consumer market, although some dairy farms in China encountered sales difficulties in the early stages of the pandemic [78,95], starting from the second quarter of 2020, Chinese dairy companies actively implemented the policy of “guarantee price, quality, and supply”. Meanwhile, taking into account the particular circumstances during the pandemic, the Chinese government proposed that “immunity is the best special medicine”, and included the slogan, “drink a glass of milk every day” in the national dietary guidelines. With the efforts of enterprises and national and local governments, dairy products became the choice of more consumers [96]. During the COVID-19 pandemic, the Chinese government issued several policies to promote the consumption of dairy products. In order to prevent and control COVID-19, the National Health Commission of China issued the “Nutritious Dietary Guidelines for the Prevention and Control of Pneumonia from New Coronavirus Infection” in 2020, encouraging people to consume a variety of milk and other dairy products, especially yogurt, and encouraged people to consume 300 g of liquid milk per day [97]. In the same year, in response to the COVID-19 pandemic, the State Council of China issued a notice on stabilizing the production and supply of agricultural products, and listed milk as a daily necessity for the first time [17]. In addition, in the “Letter on the Specific Scope of Providing Key Guarantee Materials for COVID-19 Prevention and Control” issued by the National Development and Reform Commission of China, milk was listed as one of the key guarantee foods during the pandemic [14,18].

During the SARS pandemic in 2003, many Chinese consumers made lifestyle changes in search of security, reducing the number and times of going out to public places. The COVID-19 pandemic in 2020 once again changed the lifestyle and consumption patterns of the Chinese population, and people also reduced the number and times of going out to public places. However, people’s consumption of dairy products changed from the original pattern of multiple and small purchases to the tendency to purchase less frequently and to make one-time bulk purchases [98]. Although many dairy products are perishable, most of the sales of dairy products in the Chinese market are in the form of liquid milk [27]. As early as 1997–2007, with the adoption of Tetra Pak packaging in the Chinese dairy product market, UHT milk was promoted throughout China [14]. Although the sales of low-temperature dairy products that are not shelf stable during the pandemic decreased, the sales of UHT milk increased; due to the ultra-high temperature sterilization method and the convenient storage of Tetra Pak, UHT milk became one of the important choices for Chinese consumers to stock up during the pandemic [14]. In addition, taking into account the particular circumstances during the pandemic, the Chinese government’s vigorous publicity in which the government stated that consuming dairy products can boost immunity, dairy products developed an improved image as having stronger health attributes in many consumers’ perceptions. Despite the pandemic leading, overall, to reduced spending on animal-derived foods, there was an increase in the consumption of dairy products in China. Based on the above perspectives, this study proposes the following hypothesis:

**Hypothesis 1.**  *In China*, *the COVID-19 pandemic increased the consumption of dairy products.*

## 3. Method

### 3.1. Translog Model

Barclay and Brand-Miller [99] conducted a study in Australia, using the sales data provided by beverage manufacturers, to analyze consumers’ preferences for sugar. The study found that sugar consumption has declined in the past 30 years. In a similar study, scholars used sales data to examine Canadian consumers’ consumption preferences for drinks and alcohol [100,101]. Building on this past research, we have also used sales-related data to evaluate trends in dairy consumption during the pandemic.

Several factors affect the estimation of the consumption of dairy products in China, such as production, exports, and imports. Therefore, a rough estimation of dairy consumption equals production plus imports minus exports. According to the “2021 China Dairy Industry Quality Report” issued by the Ministry of Agriculture and Rural Affairs of China [102], in 2020, China’s dairy production was 35.3 million tons. According to China Customs [103] Statistics, in 2020, China imported 3.281 million tons of dairy products, and China exported 42,940.84 tons of dairy products. Using the above figures to calculate the dairy consumption shows that the total dairy product consumption of the Chinese population in 2020 was about 38.538 million tons (3530 + 328.12 − 4.294), of which the output of Chinese dairy companies accounted for 91.49% and imported dairy products accounted for 8.51%. It can be seen from the figures that in the dairy consumption structure of the Chinese population, the vast majority of dairy products are produced by Chinese dairy companies. In addition, given the high market concentration of China’s dairy industry and the huge market share of China’s listed dairy companies [22,23,24,25,26], the sales-related data of listed Chinese dairy products companies can serve as an effective proxy for the consumption of dairy products.

The growth in dairy product sales is strongly linked to the population’s increasing consumption of dairy products from the supply-side perspective. As per basic economic theory, revenue is the product of product price and the quantity sold. In response to the COVID-19 pandemic, Chinese dairy companies actively implemented the government’s policy of ensuring dairy products’ price, quality, and supply. As a result, the prices of dairy products in China remained stable, and any changes in the sales volume of dairy products had a direct impact on the company’s revenues. On the demand side, any changes in the quantity of dairy products purchased by consumers would also affect the revenues of dairy firms. Therefore, the revenue changes in the dairy industry can provide a proxy variable for trends in China’s dairy consumption from the viewpoint of dairy companies and accounting statements. The proxy index in empirical research usually refers to the main representativeness. Our indicators are highly representative of this research and are also the most suitable indicators we can obtain, which can be used to achieve the purpose of this research.

Tsai et al. [104] reported that high-quality human resources are the basis for the stable progress of an enterprise. In China, the dairy industry is composed of a variety of human resources, including people engaged as management personnel, research and development professionals, and ordinary employees. The production function of the dairy industry is as follows:

(1) $$y=f\left(x_{1},x_{2},x_{3},f,\;d,\;b\right)$$

*y* is the overall sales of dairy products; $x_{1}$ represents the number of personnel input for management; $x_{2}$ represents the number of personnel input for research and development; and $x_{3}$ represents the number of personnel input for ordinary employees. *f*, *d*, and *b*, respectively, represent the amount of investment in fixed assets, research and development (R&D), and productive biological assets. According to economic theory, on the one hand, input must be greater than 0: $y,x_{i},f,\;d\geq 0$, and *i* = 1, 2, 3; on the other hand, these theoretical variables need to meet the following conditions: $\partial^{2}f(\cdot )/\partial x_{i}^{2}\leq 0$, $\partial^{2}f(\cdot )/\partial f^{2}\leq 0$, $\partial^{2}f(\cdot )/\partial d^{2}\leq 0$, and $\partial^{2}f(\cdot )/\partial b^{2}\leq 0$.

This study uses the following equation to express the revenue function of the dairy industry:

(2) $$r\left(p;x_{1},x_{2},x_{3},f,\;d,\;b\right)=max\;py\;\mathrm{subject}\mathrm{to}y=f\left(x_{1},x_{2},x_{3},f,\;d,\;b\right)$$

In Equation (2), *r* is the revenue of the dairy industry, and *p* is the price of dairy products. In research, the Cobb–Douglas function is commonly used to convert the above equation. Equation (3) represents the converted revenue function:

(3) $$\ln r=\alpha_{0}+\delta \ln p+\sum_{i=1}^{3}\alpha_{i}\ln x_{i}+\beta_{1}\ln f+\delta_{1}ln\;d+\epsilon_{1}ln\;b$$

The output of the above model is a single output, and the important feature of this model is that of homogeneous degree 1 in the output price (δ = 1). We normalize the model by *p =* 1 [105] and express it using Equation (4):

(4) $$\ln r=\alpha_{0}+\sum_{i=1}^{3}\alpha_{i}\;ln\;x_{i}+\beta_{1}ln\;f+\delta_{1}ln\;d+\epsilon_{1}ln\;b$$

We further draw on the research methods of previous scholars on the translog revenue function model [105], and specify the function model of the dairy industry in Equation (5), as follows [106]:

(5) $$\begin{array}{cc} ln\;r=\alpha_{0}+\sum_{i=1}^{3} & \alpha_{i}\;ln\;x_{i}+\beta_{1}ln\;f+\delta_{1}ln\;d+\epsilon_{1}ln\;b+\frac{1}{2}\sum_{i=1}^{3}\sum_{l=1}^{3}\alpha_{il}\;ln\;x_{i}\;ln\;x_{l}+\frac{1}{2}\beta_{11}{\left(\ln f\right)}^{2}+\frac{1}{2}\delta_{11}{\left(\ln d\right)}^{2}\\ & +\frac{1}{2}\epsilon_{11}{\left(\ln b\right)}^{2}+\sum_{i=1}^{3}\gamma_{i1}{\ln x}_{i}ln\;f+\sum_{i=1}^{3}\varepsilon_{i1}{\ln x}_{i}ln\;d+\sum_{i=1}^{3}\mu_{i1}{\ln x}_{i}ln\;b+\theta_{11}ln\;f\;ln\;d\\ & +\rho_{11}ln\;f\;ln\;b+\sigma_{11}ln\;d\;ln\;b \end{array}$$

In this study, if $\alpha_{il}=\beta_{11}=\delta_{11}=\epsilon_{11}=\gamma_{i1}=\varepsilon_{i1}=\mu_{i1}=\theta_{11}=\rho_{11}=\sigma_{11}=0$, the equation will return to the Cobb–Douglas function (log-linear specification). 

### 3.2. Selection of Variables and Samples

#### 3.2.1. How to Choose Samples

This study focuses on the changes in dairy consumption in the year the pandemic began, so our data focused on years starting in 2020 and before. The largest pool of listed companies in China, the CSMAR database, was used in a comprehensive analysis of the revenue and input variables of China’s dairy industry between 2016 and 2020. To ensure data integrity, we excluded some unreasonable data, such as dairy companies with zero total revenue, zero employees across all categories, zero fixed assets, zero productive biological assets, zero R&D investment, and so on. Ultimately, a quarterly dataset comprising 148 valid observations was obtained.

#### 3.2.2. Variable Definitions

We included multiple inputs from the dairy industry on the right-hand side of the model, while the total revenue of the dairy industry (*DREVENUE*) serves as the dependent variable on the left-hand side. To measure personnel input, we employed a set of proxy variables, namely, management personnel (*MSTAFF*), R&D professionals (*RSTAFF*), and ordinary employees (*OSTAFF*). Furthermore, we obtained the values of fixed assets (*FIXED*), R&D investment (*DEVELOP*), and productive biological assets (*BIOLOGY*) from the financial statements of the listed dairy companies to represent the corresponding asset inputs, respectively.

This study considered an additional dummy variable called *BIG*, which represents China’s top three largest dairy products companies with a very high market share. *BIG* has a complete online ordering system and convenient door-to-door service. In the context of the pandemic, *BIG* has addressed the problem of people going out less because of traffic restrictions to a significant extent. The consumption of dairy products produced by *BIG* and non-*BIG* enterprises differs. Therefore, this study adds the top three dairy product companies (*BIG*) as a dummy variable. If a dairy product company belongs to China’s top three dairy product companies, then *BIG* equals 1; otherwise, it equals 0. Table 1 provides detailed definitions for all variables examined in this study. We referred to the study of Coelli et al. [107] and added the annual variable (*YEAR*) to the model to control the influence of different factors in different years and to cover the entire economic growth through the *YEAR* variable. The addition of the *YEAR* variable in the econometric treatment reflects the changes in the industry over time and technology and the supply and demand sides of the industry. If the demand side of the enterprise increases with the economic growth, the supply side will also change. These factors are all reflected in the *YEAR* variable, including the impact of economic development, changes in GNP, changes in GDP, an increase in the number of dairy enterprises, economic recession, and other factors, which are all included in the annual factors. 

After excluding other factors through the *YEAR* variable, we added a *COVID* dummy variable to our analysis to assess the effect of the COVID-19 pandemic on the dairy products market. When *COVID* equals 1, it represents data for 2020; when *COVID* equals 0, this means data for the four years from 2016 to 2019.

### 3.3. Estimation Model

In this study, the theoretical model was rewritten into an estimation model, and the relationship between *DREVENUE*, *MSTAFF*, *RSTAFF*, *OSTAFF*, *FIXED*, *DEVELOP*, and *BIOLOGY* inputs in the dairy industry was represented by the translog revenue function. *COVID* had a significant influence on the relationship among these variables. This study used annual pooling data to calculate the model. We added *BIG* and *YEAR* to the model. Next, we rewrote Equation (5):

(6) $$\begin{array}{cc} \ln DREVENUE= & \alpha_{0}+\alpha_{1}\ln MSTAFF+\alpha_{2}\ln RSTAFF+\alpha_{3}\ln OSTAFF+\beta_{1}\ln FIXED+\delta_{1}\ln DEVELOP\\ & +\epsilon_{1}lnBIOLOGY+\frac{1}{2}\;\alpha_{11}\;{\left(ln\;MSTAFF\right)}^{2}+\frac{1}{2}\;\alpha_{22}\;{\left(ln\;RSTAFF\right)}^{2}+\frac{1}{2}\;\alpha_{33}\;{\left(ln\;OSTAFF\right)}^{2}\\ & +\frac{1}{2}\;\beta_{11}\;{\left(ln\;FIXED\right)}^{2}+\frac{1}{2}\;\delta_{11}\;{\left(ln\;DEVELOP\right)}^{2}+\frac{1}{2}\;\epsilon_{11}\;{\left(ln\;BIOLOGY\right)}^{2}\\ & +\alpha_{12}\ln MSTAFF\ln RSTAFF+\alpha_{13}\ln MSTAFF\ln OSTAFF+\alpha_{23}\;ln\;RSTAFF\;ln\;OSTAFF\\ & +\gamma_{11}\ln MSTAFF\ln FIXED+\gamma_{21}\;ln\;RSTAFF\;ln\;FIXED+\gamma_{31}\;ln\;OSTAFF\;ln\;FIXED\\ & +\varepsilon_{11}\ln MSTAFF\ln DEVELOP+\varepsilon_{21}ln\;RSTAFF\;ln\;DEVELOP+\varepsilon_{31}\;ln\;OSTAFF\;ln\;DEVELOP\\ & +\mu_{11}\ln MSTAFF\ln BIOLOGY+\mu_{21}\ln RSTAFF\ln BIOLOGY+\mu_{31}\;ln\;OSTAFF\ln BIOLOGY\\ & +\theta_{11}\;ln\;FIXED\;ln\;DEVELOP+\rho_{11}\;ln\;FIXED\;ln\;BIOLOGY+\sigma_{11}\;ln\;DEVELOP\;ln\;BIOLOGY\\ & +\varphi_{1}\;BIG+\varphi_{2}\;COVID+\varphi_{3}\;BIG\;COVID+\tau_{1}\;YEAR+\tau_{2}\;{YEAR}^{2} \end{array}$$

Equation (6) is the core model of this study. Through Equation (6) and the proxy variables of this research, we can calculate the following equations, including the average partial effect of various inputs.

The average partial effect (APE) of *MSTAFF* on *DREVENUE:*

(7) $$\begin{array}{cc} \partial \hat{ln\;DREVENUE}/ & \partial ln\;MSTAFF\\ & ={\hat{\alpha }}_{1}+{\hat{\alpha }}_{11}\bar{ln\;MSTAFF}+{\hat{\alpha }}_{12}\bar{ln\;RSTAFF}+{\hat{\alpha }}_{13}\bar{ln\;OSTAFF}\\ & +{\hat{\gamma }}_{11}\bar{ln\;FIXED}+{\hat{\varepsilon }}_{11}\bar{ln\;DEVELOP}+{\hat{\mu }}_{11}\bar{ln\;BIOLOGY} \end{array}$$

The APE of *RSTAFF* on *DREVENUE*:

(8) $$\begin{array}{cc} \partial \hat{ln\;DREVENUE}/ & \partial ln\;RSTAFF\\ & ={\hat{\alpha }}_{2}+{\hat{\alpha }}_{22}\bar{ln\;RSTAFF}+{\hat{\alpha }}_{12}\bar{ln\;MSTAFF}+{\hat{\alpha }}_{23}\bar{ln\;OSTAFF}\\ & +{\hat{\gamma }}_{21}\bar{ln\;FIXED}+{\hat{\varepsilon }}_{21}\bar{ln\;DEVELOP}+{\hat{\mu }}_{21}\bar{ln\;BIOLOGY} \end{array}$$

The APE of *OSTAFF* on *DREVENUE*:

(9) $$\begin{array}{cc} \partial \;\hat{ln\;DREVENUE}/ & \partial \;ln\;OSTAFF\\ & ={\hat{\alpha }}_{3}+{\hat{\alpha }}_{33}\bar{ln\;OSTAFF}+{\hat{\alpha }}_{13}\bar{ln\;MSTAFF}+{\hat{\alpha }}_{23}\bar{ln\;RSTAFF}\\ & +{\hat{\gamma }}_{31}\bar{ln\;FIXED}+{\hat{\varepsilon }}_{31}\bar{ln\;DEVELOP}+{\hat{\mu }}_{31}\bar{ln\;BIOLOGY} \end{array}$$

The APE of *FIXED* on *DREVENUE*:

(10) $$\begin{array}{cc} \partial \hat{ln\;DREVENUE}/ & \partial ln\;FIXED\\ & ={\hat{\beta }}_{1}+{\hat{\beta }}_{11}\bar{ln\;FIXED}+{\hat{\gamma }}_{11}\bar{ln\;MSTAFF}+{\hat{\gamma }}_{21}\bar{ln\;RSTAFF}\\ & +{\hat{\gamma }}_{31}\bar{ln\;OSTAFF}+{\hat{\theta }}_{11}\bar{lnDEVELOP}+{\hat{\rho }}_{11}\bar{ln\;BIOLOGY} \end{array}$$

The APE of *DEVELOP* on *DREVENUE*:

(11) $$\begin{array}{cc} \partial \hat{ln\;DREVENUE}/ & \partial ln\;DEVELOP\\ & ={\hat{\delta }}_{1}+{\hat{\delta }}_{11}\bar{ln\;DEVELOP}+{\hat{\varepsilon }}_{11}\bar{ln\;MSTAFF}+{\hat{\varepsilon }}_{21}\bar{ln\;RSTAFF}\\ & +{\hat{\varepsilon }}_{31}\bar{ln\;OSTAFF}+{\hat{\theta }}_{11}\bar{ln\;FIXED}+{\hat{\sigma }}_{11}\bar{ln\;BIOLOGY} \end{array}$$

The APE of *BIOLOGY* on *DREVENUE*:

(12) $$\begin{array}{l} \partial \hat{ln\;DREVENUE}/\partial ln\;BIOLOGY\\ \;\;\;\;\;\;\;\;\;\;\;\;={\hat{\epsilon }}_{1}+{\hat{\epsilon }}_{11}\bar{ln\;BIOLOGY}+{\hat{\mu }}_{11}\bar{ln\;MSTAFF}+{\hat{\mu }}_{21}\bar{ln\;RSTAFF}\\ \;\;\;\;\;\;\;\;\;\;\;\;+{\hat{\mu }}_{31}\bar{ln\;OSTAFF}+{\hat{\rho }}_{11}\bar{ln\;FIXED}+{\hat{\sigma }}_{11}\bar{ln\;DEVELOP} \end{array}$$

The APE of *BIG* on *DREVENUE:*

(13) $$\partial \hat{ln\;DREVENUE}/\partial BIG=\varphi_{1}+\varphi_{3}\;COVID$$

The APE of *COVID* on *DREVENUE*:

(14) $$\partial \hat{ln\;DREVENUE}/\partial COVID=\varphi_{2}+\varphi_{3}\;BIG$$

The APE of *YEAR* on *DREVENUE*:

(15) $$\partial \hat{ln\;DREVENUE}/\partial YEAR=\tau_{1}+2*\tau_{2}\;\bar{YEAR}$$

## 4. Results

### 4.1. Descriptive Statistics and Correlation Matrix

Table 2 depicts the statistical information on China’s dairy industry data from 2016 to 2020. It can be seen from Table 2 that in all years of the study period, most of the average values of *DREVENUE*, *RSTAFF*, *OSTAFF*, *FIXED*, and *BIOLOGY* of the dairy industry are greater than the median. This infers that the overall research data are tilted to the right. The standard deviation of *DREVENUE* from 2016 to 2020 is at a relatively high level, which means that, as a whole, the scale of different companies in the dairy industry varies greatly. The average revenue of the dairy industry showed an overall upward trend from 2016 to 2019, rising from CNY 3400 million in 2016 to RMB 3840 million in 2020. This remarkable growth of approximately 12.94% over five years underscores Chinese consumers’ escalating consumption of dairy products. This may also be related to inflation, population growth or the pricing of dairy products. In addition, from 2016 to 2020, the overall research and development investment (*DEVELOP*) of dairy products companies showed an upward trend, with *DEVELOP* investment rising by 38.49%, which shows that Chinese dairy enterprises attached importance to research and development. From the data in 2020, it can be seen that as a result of the pandemic’s impact, dairy companies were still actively investing in research and development, alongside continued expansion in *FIXED*, *BIOLOGY*, *MSTAFF*, *RSTAFF*, and *OSTAFF*. This indicates a prevailing confidence within China’s dairy industry regarding the prospects of the consumer market.

According to the study, the correlation coefficients for both Spearman and Pearson are presented in Table 3. The values in Table 3 indicate that *DREVENUE* and *COVID* are positively correlated. The possible reasons are (1) the Chinese government’s propaganda on enhancing immunity by consuming dairy products during the pandemic, (2) the Chinese government and enterprises’ guarantee of the price stability of dairy products, and (3) that Chinese citizens were paying more attention to health during the pandemic. The above reasons have resulted in consumers tending to adopt a consumption strategy of one-time bulk purchases to hoard dairy products to a certain extent. Consumers’ increased consumption of dairy products has led to an increase in the overall revenue for the dairy industry. *COVID* has demonstrated a significant positive correlation with *OSTAFF*, *FIXED*, *DEVELOP*, and *BIOLOGY*. This shows that Chinese dairy companies generally continued to increase these investments even during the pandemic.

### 4.2. Test Whether the Pandemic Has an Impact on Dairy Consumption Trends

The objective of our research is to investigate the impact of the COVID-19 pandemic on dairy consumption. With the outbreak of the pandemic in 2020 as the cut-off point, we estimated dairy industry revenue using the Chow Test, and the F statistic measured was equal to 2.82, rejecting the null hypothesis. This study uses its results to prove that the COVID-19 pandemic affected the market for dairy products. On the basis of the above results, we added *COVID* as a dummy variable to the translog revenue function to evaluate the specific impact of COVID-19 on the market for dairy products. Furthermore, we incorporated the use of *BIG* to analyze the differential impact of the pandemic on dairy products sourced from various origins within the dairy products market.

### 4.3. Estimation Results

#### 4.3.1. Study Model Validation

To ensure that the study results are accurately represented, the translog revenue function was used instead of the Cobb–Douglas function in this study. The translog function offers several advantages that are better suited to this study. The empirical results of this study are presented in Table 4.

However, it is crucial to ascertain the accuracy of whether the translog revenue function effectively represents the obtained results. Therefore, Equation (6) needs to be tested, as follows:

(16) $$\alpha_{il}=\beta_{11}=\delta_{11}=\epsilon_{11}=\gamma_{i1}=\varepsilon_{i1}=\mu_{i1}=\theta_{11}=\rho_{11}=\sigma_{11}=0\;for\;all\;i=1,2,3.$$

According to the above equation, the F statistic for the given model is 2.75, as reported in Table 4. Therefore, it was determined that the translog revenue function is a better approach to assess the impact of the pandemic on dairy consumption, compared to the Cobb–Douglas function and based on the test results.

#### 4.3.2. Dairy Products Consumption Trends during the COVID-19 Pandemic

Table 5 shows that the average partial effect of *YEAR* on *DREVENUE* is positive and significant, indicating that consumers increase their consumption of dairy products with economic growth, technological changes, and an increase in the number of enterprises and other factors. After excluding other factors through the *YEAR* variable, based on the data presented in Table 5, the average partial effect of *COVID* on *DREVENUE* is positive and significant. According to Table 4, the translog model shows a significant positive coefficient of *COVID*. This indicates increased dairy product consumption in China due to the COVID-19 pandemic. Therefore, Hypothesis 1 is supported by the data.

**Figure 1:**
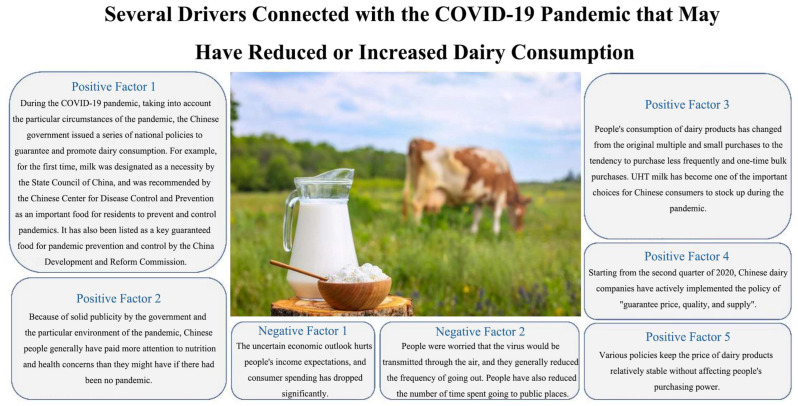
Several drivers connected with the COVID-19 pandemic that may have reduced or increased dairy consumption.

We analyzed the possible reasons for this increase, taking into account the particular circumstances during the pandemic when the Chinese government issued several policies to promote people’s consumption of dairy products. Because of the government’s strong publicity campaign and the special environment created by the pandemic, Chinese people generally paid more attention to nutritional and health concerns than they might have if there had been no pandemic. As a result of the challenges associated with transportation caused by the pandemic, Chinese consumers were more inclined to hoard dairy products (mainly UHT milk) through one-time bulk purchases [14]. Another reason that cannot be ignored is that although people’s concerns about their own uncertain economic future intensified, Chinese dairy companies actively implemented the policy of “guarantee price, quality, and supply”, making the price of dairy products relatively stable without affecting people’s purchasing power. Figure 1 lists several drivers connected with the COVID-19 pandemic that may have reduced or increased dairy consumption. It is clear that the drivers of increasing dairy consumption were more robust in China.

The results presented in Table 5 demonstrate that the APE of *COVID* for non-*BIG* enterprises (0.628) is comparatively smaller than the *APE* of *COVID* for *BIG* enterprises (1.464). This suggests a higher inclination among consumers to purchase dairy products from larger enterprises during the pandemic. A possible reason for this is that larger dairy companies differ from small and medium-sized dairy companies. Larger dairy companies have a broader sales network, more complete online sales channels, and door-to-door services, more robust and resilient supply chains, and will have priority when it comes to sourcing and distribution. As a result, the consumption of dairy products produced by large enterprises rose more during the pandemic. Through an in-depth analysis of the pandemic’s impact mechanism on consumption, we found that people’s restrictions on going out and their fear of the virus during the pandemic are important reasons why people may change their consumption patterns. Large dairy enterprises can provide convenience and safety for consumers to a large extent, through the mechanisms of online sales and door-to-door delivery.

## 5. Conclusions

### 5.1. Discussions and Suggestions

The COVID-19 pandemic significantly impacted consumers’ food spending due to widespread social distancing measures, lockdowns, business closures, and the economic insecurity that preceded the post-pandemic era. This global health crisis also posed substantial challenges for the dairy cattle sector while impacting consumption trends of dairy products. However, the current, related study is relatively limited. Tesfaye et al. [7] and Hirvonen et al. [6] reported that people have also been affected by the fear of the virus since the pandemic and have reduced their frequency of going out. In Ethiopia, households consuming dairy products have declined 11 percentage points. A previous study conducted by Ceballos et al. [11] found that the pandemic led to a decrease in the consumption of dairy products in rural Guatemala. 

However, in developed countries such as the United States, studies have reported increased dairy consumption during the pandemic [108]. In the US, lockdowns and restaurant closures led to an increase in people cooking at home, which boosted the consumption of dairy products such as butter and liquid milk. The consumption of butter increased by more than 3% year on year, and the consumption of liquid milk increased by more than 2% year on year. Additionally, 70% of consumers indicated that they planned to buy the same amount of refrigerated dairy, cheese, and butter during the pandemic; 20% planned to buy more cheese and yogurt [108]. During the pandemic, the online purchase of dairy products increased unprecedentedly. Compared with the same period in 2019, from March to August 2020, the number of households where consumers bought butter and milk online increased by more than 200% [108]. US dairy product consumption has increased due to increased cheese and butter consumption. Our study found some similarities between dairy product consumption trends in China and the United States.

Based on economic theory and accounting data from the dairy cattle sector, our findings reveal that COVID-19 resulted in a significant surge in the consumption of dairy products in China. However, we also identified some challenges that need to be addressed. The increase in dairy consumption during the pandemic may be attributed to the policies implemented by the Chinese government to encourage the consumption of dairy products, in particular, in light of the unique circumstances of the pandemic. It may also be related to the Chinese dairy cattle sector’s active implementation of the “guarantee price, quality, and supply” policy. People generally paid more attention to nutrition during the pandemic, and there was an increase in the number of meals prepared and eaten at home.

Despite the surge in overall dairy product consumption in China attributed to the COVID-19 pandemic, it is imperative to acknowledge the concurrent societal challenges precipitated by this global health crisis. When people face economic uncertainty, such as during a pandemic, they often reduce food expenditure (especially those at the bottom of the income pyramid). They also tend to save more money, which reduces Engel’s coefficient. Any major crisis will profoundly impact the lower strata of the income pyramid, leading to a reduction in nutritional intake among this population segment. Consequently, despite an overall increase in dairy product consumption within society as a whole during the pandemic, low-income groups in China, particularly vulnerable during pandemics, may have encountered challenges in accessing adequate quantities of dairy and other food products. Urgent government support is imperative in crisis situations to ensure nutritional security for this economically disadvantaged population.

Based on the above societal challenges, we put forward these suggestions. The government can consider starting from the following two aspects: First, the COVID-19 pandemic significantly impacted the disposable income of the low-income segment of the population. The government may consider providing this group of people with emergency purchasing subsidies for dairy products and other foods to provide nutritional protection for the low-income segment of the population during a pandemic. Second, the government can consider directly distributing dairy products and other nutrients to low-income families or individuals in any major public health emergency. In addition, to provide sufficient quantities of dairy products to the poorer groups in society, dairy products could be included in the ration plan for a significant period of time. Meanwhile, in terms of enterprises, dairy companies could consider cooperating with organizations such as the Red Cross and Hope Primary Schools to donate or sell dairy products at low prices, thus allowing people in poverty to obtain more nutritional protection during pandemics.

### 5.2. Limitations and Future Studies

This study examined the effects of the COVID-19 pandemic on dairy consumption from the perspective of the Chinese dairy cattle sector, focusing on the year when the pandemic began by comparing data from previous years. Although the import and export volume of dairy products has not reached 10% of the total consumption, if scholars can obtain detailed data on the input, output, and sales of the imports and exports of dairy product companies, this area of study should be able to obtain more accurate results.

## Figures and Tables

**Table 1:** Definition of all variables.

Variable		Definition
Theoretical Variable	Proxy Variable	
*r*	*DREVENUE*	The revenue of dairy enterprise
*x*_1_	*MSTAFF*	Number of all management employees
*x*_2_	*RSTAFF*	Number of R&D employees
*x*_3_	*OSTAFF*	Number of researchers and developers
	*EMPLOYEE*	Number of employees of all types
*f*	*FIXED*	Ending balance of fixed assets
*d*	*DEVELOP*	R&D expenses
*b*	*BIOLOGY*	Ending balance of productive biological assets
	*BIG*	*BIG* is a dummy variable. If the dairy product enterprise is not in the top three dairy product enterprises in China, *BIG* is equal to 0; otherwise, it is equal to 1
	*COVID*	*COVID* is a dummy variable. If the year is between 2016 and 2019, *COVID* is equal to 0; otherwise, it is equal to 1
	*YEAR*	In 2016, *YEAR* is equal to 1; in 2017, *YEAR* is equal to 2, and so on

**Table 2:** Descriptive statistics (the unit of *DREVENUE*, *FIXED*, *DEVELOP*, and *BIOLOGY* is CNY million).

	**2016 (*n* = 29)**					**2017 (*n* = 29)**				
**Variables**	**Max**	**Min**	**Mean**	**Median**	**Std. Dev.**	**Max**	**Min**	**Mean**	**Median**	**Std. Dev.**
*DREVENUE*	CNY 20,200.00	CNY 141.00	CNY 3400.00	CNY 1100.00	CNY 5220.00	CNY 22,000.00	CNY 154.00	CNY 3120.00	CNY 1240.00	CNY 5080.00
*MSTAFF*	18.00	13.00	16.00	17.00	1.60	17.00	13.00	15.45	16.00	1.40
*RSTAFF*	346.00	8.00	96.21	65.00	86.21	218.00	6.00	114.14	86.00	81.36
*OSTAFF*	54,621.00	1035.00	4940.17	1674.00	9828.42	7985.00	702.00	3154.97	1864.00	2526.24
*EMPLOYEE*	54,983.00	1255.00	5052.38	1879.00	9870.14	8055.00	724.00	3284.55	2075.00	2487.43
*FIXED*	CNY 14,700.00	CNY 261.00	CNY 2020.00	CNY 647.00	CNY 3010.00	CNY 6120.00	CNY 257.00	CNY 1700.00	CNY 686.00	CNY 1850.00
*DEVELOP*	CNY 172.00	CNY 1.68	CNY 33.23	CNY 30.94	CNY 30.25	CNY 49.51	CNY 1.44	CNY 28.93	CNY 34.61	CNY 15.95
*BIOLOGY*	CNY 1310.00	CNY 29.66	CNY 273.00	CNY 75.50	CNY 397.00	CNY 1160.00	CNY 30.93	CNY 250.00	CNY 83.46	CNY 356.00
	**2018 (*n* = 31)**					**2019 (*n* = 30)**				
**Variables**	**Max**	**Min**	**Mean**	**Median**	**Std. Dev.**	**Max**	**Min**	**Mean**	**Median**	**Std. Dev.**
*DREVENUE*	CNY 21,000.00	CNY 147.00	CNY 3100.00	CNY 1230.00	CNY 4760.00	CNY 22,600.00	CNY 193.00	CNY 3580.00	CNY 1380.00	CNY 5180.00
*MSTAFF*	17.00	10.00	13.45	15.00	2.61	17.00	12.00	14.53	14.00	1.74
*RSTAFF*	215.00	15.00	95.61	55.00	73.45	214.00	10.00	99.20	100.00	72.19
*OSTAFF*	12,662.00	494.00	3890.19	2004.00	4132.86	12,013.00	873.00	4366.60	2051.00	3855.84
*EMPLOYEE*	12,765.00	658.00	3999.26	2036.00	4121.06	12,125.00	922.00	4480.33	2078.00	3851.63
*FIXED*	CNY 5950.00	CNY 563.00	CNY 1760.00	CNY 853.00	CNY 1730.00	CNY 7590.00	CNY 813.00	CNY 2030.00	CNY 1090.00	CNY 1920.00
*DEVELOP*	CNY 64.59	CNY 3.91	CNY 28.17	CNY 28.73	CNY 19.45	CNY 69.75	CNY 3.18	CNY 38.14	CNY 47.74	CNY 26.29
*BIOLOGY*	CNY 1080.00	CNY 34.41	CNY 265.00	CNY 93.73	CNY 327.00	CNY 966.00	CNY 36.10	CNY 290.00	CNY 262.00	CNY 282.00
	**2020 (*n* = 29)**					**ALL (*n* = 148)**				
**Variables**	**Max**	**Min**	**Mean**	**Median**	**Std. Dev.**	**Max**	**Min**	**Mean**	**Median**	**Std. Dev.**
*DREVENUE*	CNY 25,200.00	CNY 141.00	CNY 3840.00	CNY 1640.00	CNY 5770.00	CNY 25,200.00	CNY 141.00	CNY 3410.00	CNY 1260.00	CNY 5140.00
*MSTAFF*	19.00	11.00	14.72	15.00	2.46	19.00	10.00	14.81	15.00	2.18
*RSTAFF*	190.00	13.00	104.69	94.00	69.97	346.00	6.00	101.86	76.00	76.06
*OSTAFF*	11,750.00	1241.00	4843.97	2195.00	3890.47	54,621.00	494.00	4235.32	2051.00	5417.29
*EMPLOYEE*	11,856.00	1268.00	4963.38	2348.00	3894.63	54,983.00	658.00	4352.00	2078.00	5425.99
*FIXED*	CNY 8370.00	CNY 710.00	CNY 2270.00	CNY 1190.00	CNY 2320.00	CNY 14,700.00	CNY 257.00	CNY 1950.00	CNY 951.00	CNY 2190.00
*DEVELOP*	CNY 74.97	CNY 5.22	CNY 46.02	CNY 51.89	CNY 28.07	CNY 172.00	CNY 1.44	CNY 34.83	CNY 34.61	CNY 25.10
*BIOLOGY*	CNY 851.00	CNY 36.23	CNY 346.00	CNY 383.00	CNY 282.00	CNY 1310.00	CNY 29.66	CNY 285.00	CNY 118.00	CNY 329.00

Note: Please refer to Table 1 for the definitions of the variables.

**Table 3:** The Spearman and Pearson Correlation Coefficients.

	*DREVENUE*	*MSTAFF*	*RSTAFF*	*OSTAFF*	*EMPLOYEE*	*FIXED*	*DEVELOP*	*BIOLOGY*	*BIG*	*COVID*
*DREVENUE*	1.000	−0.329 ***	−0.02	0.589 ***	0.588 ***	0.847 ***	0.488 ***	0.721 ***	0.597 ***	0.102
	-----	(0.000)	(0.809)	(0.000)	(0.000)	(0.000)	(0.000)	(0.000)	(0.000)	(0.217)
*MSTAFF*	−0.268 ***	1.000	−0.036	−0.178 **	−0.178 **	−0.36 ***	−0.359 ***	−0.351 ***	−0.257 ***	−0.025
	(0.001)	-----	(0.662)	(0.030)	(0.031)	(0.000)	(0.000)	(0.000)	(0.002)	(0.765)
*RSTAFF*	0.061	−0.096	1.000	0.113	0.126	0.022	0.543 ***	−0.091	−0.196 **	0.02
	(0.458)	(0.248)	-----	(0.173)	(0.126)	(0.789)	(0.000)	(0.269)	(0.017)	(0.807)
*OSTAFF*	0.807 ***	−0.247 ***	−0.135	1.000	1.000 ***	0.836 ***	0.668 ***	0.527 ***	0.607 ***	0.047
	(0.000)	(0.002)	(0.102)	-----	(0.000)	(0.000)	(0.000)	(0.000)	(0.000)	(0.574)
*EMPLOYEE*	0.817 ***	−0.243 ***	−0.052	0.994 ***	1.000	0.835 ***	0.675 ***	0.524 ***	0.603 ***	0.047
	(0.000)	(0.003)	(0.533)	(0.000)	-----	(0.000)	(0.000)	(0.000)	(0.000)	(0.572)
*FIXED*	0.767 ***	−0.443 ***	−0.056	0.811 ***	0.806 ***	1.000	0.631 ***	0.806 ***	0.665 ***	0.065
	(0.000)	(0.000)	(0.497)	(0.000)	(0.000)	-----	(0.000)	(0.000)	(0.000)	(0.432)
*DEVELOP*	0.506 ***	−0.446 ***	0.624 ***	0.451 ***	0.493 ***	0.493 ***	1.000	0.387 ***	0.467 ***	0.186 **
	(0.000)	(0.000)	(0.000)	(0.000)	(0.000)	(0.000)	-----	(0.000)	(0.000)	(0.024)
*BIOLOGY*	0.089	−0.218 ***	−0.057	0.031	0.011	0.185 **	0.14 *	1.000	0.378 ***	0.087
	(0.281)	(0.008)	(0.493)	(0.711)	(0.890)	(0.025)	(0.090)	-----	(0.000)	(0.293)
*BIG*	0.755 ***	−0.290 ***	−0.059	0.844 ***	0.84 ***	0.845 ***	0.464 ***	−0.024	1.000	0.003
	(0.000)	(0.000)	(0.476)	(0.000)	(0.000)	(0.000)	(0.000)	(0.774)	-----	(0.970)
*COVID*	0.167 **	−0.053	0.005	0.139 *	0.128	0.152 *	0.207 **	0.152 *	0.003	1.000
	(0.043)	(0.526)	(0.955)	(0.093)	(0.121)	(0.064)	(0.012)	(0.066)	(0.970)	-----

Note: Spearman’s coefficient on the left, Pearson’s coefficient on the right; Please refer to Table 1 for the definitions of the variables; The significance levels are denoted by *, **, and *** representing the 10%, 5%, and 1% levels of significance, respectively.

**Table 4:** Translog model estimates.

$\begin{array}{cc} \ln DREVENUE= & \alpha_{0}+\alpha_{1}\ln MSTAFF+\alpha_{2}\ln RSTAFF+\alpha_{3}\ln OSTAFF+\beta_{1}\ln FIXED+\delta_{1}\ln DEVELOP\\ & +\epsilon_{1}lnBIOLOGY+\frac{1}{2}\;\alpha_{11}\;{\left(ln\;MSTAFF\right)}^{2}+\frac{1}{2}\;\alpha_{22}\;{\left(ln\;RSTAFF\right)}^{2}+\frac{1}{2}\;\alpha_{33}\;{\left(ln\;OSTAFF\right)}^{2}\\ & +\frac{1}{2}\;\beta_{11}\;{\left(ln\;FIXED\right)}^{2}+\frac{1}{2}\;\delta_{11}\;{\left(ln\;DEVELOP\right)}^{2}+\frac{1}{2}\;\epsilon_{11}\;{\left(ln\;BIOLOGY\right)}^{2}\\ & +\alpha_{12}\ln MSTAFF\ln RSTAFF+\alpha_{13}\ln MSTAFF\ln OSTAFF+\alpha_{23}\;ln\;RSTAFF\;ln\;OSTAFF\\ & +\gamma_{11}\ln MSTAFF\ln FIXED+\gamma_{21}\;ln\;RSTAFF\;ln\;FIXED+\gamma_{31}\;ln\;OSTAFF\;ln\;FIXED\\ & +\varepsilon_{11}\ln MSTAFF\ln DEVELOP+\varepsilon_{21}ln\;RSTAFF\;ln\;DEVELOP+\varepsilon_{31}\;ln\;OSTAFF\;ln\;DEVELOP\\ & +\mu_{11}\ln MSTAFF\ln BIOLOGY+\mu_{21}\ln RSTAFF\ln BIOLOGY+\mu_{31}\;ln\;OSTAFF\ln BIOLOGY\\ & +\theta_{11}\;ln\;FIXED\;ln\;DEVELOP+\rho_{11}\;ln\;FIXED\;ln\;BIOLOGY+\sigma_{11}\;ln\;DEVELOP\;ln\;BIOLOGY\\ & +\varphi_{1}\;BIG+\varphi_{2}\;COVID+\varphi_{3}\;BIG\;COVID+\tau_{1}\;YEAR+\tau_{2}\;{YEAR}^{2} \end{array}$			(6)
**Variable**	**Coeff**	**Variable**	**Coeff**
	***t*-Stat.**		***t*-Stat.**
Intercept	570.664	(ln*MSTAFF*)(ln*DEVELOP*)	−1.356
	(2.755)		(−1.169)
ln*MSTAFF*	−13.296	(ln*MSTAFF*)(ln*BIOLOGY*)	−1.035
	(−0.407)		(−1.443)
ln*RSTAFF*	16.054 *	(ln*RSTAFF*)(ln*OSTAFF*)	0.904 **
	(1.779)		(2.532)
ln*OSTAFF*	29.895 **	(ln*RSTAFF*)(ln*FIXED*)	−1.432 ***
	(2.264)		(−2.945)
ln*FIXED*	−76.921 ***	(ln*RSTAFF*)(ln*DEVELOP*)	0.705 **
	(−3.690)		(2.049)
ln*DEVELOP*	15.984	(ln*RSTAFF*)(ln*BIOLOGY*)	−0.199
	(1.595)		(−0.702)
ln*BIOLOGY*	0.292	(ln*OSTAFF*)(ln*FIXED*)	−2.182 ***
	(0.064)		(−3.472)
(ln*MSTAFF*)^2^	1.208	(ln*OSTAFF*)(ln*DEVELOP*)	0.157
	(0.520)		(0.388)
(ln*RSTAFF*)^2^	−0.294	(ln*OSTAFF*)(ln*BIOLOGY*)	0.238
	(−1.262)		(1.106)
(ln*OSTAFF*)^2^	0.447	(ln*FIXED*)(ln*DEVELOP*)	−0.455
	(2.612)		(−1.091)
(ln*FIXED*)^2^	2.653 ***	(ln*FIXED*)(ln*BIOLOGY*)	−0.524
	(4.548)		(−1.545)
(ln*DEVELOP*)^2^	−0.189	(ln*DEVELOP*)(ln*BIOLOGY*)	−0.071
	(−1.187)		(−0.088)
(ln*BIOLOGY*)^2^	0.352 ***	*BIG*	1.657
	(3.326)		(1.390)
(ln*MSTAFF*)(ln*RSTAFF*)	0.714	*COVID*	0.628 **
	(0.689)		(2.155)
(ln*MSTAFF*)(ln*OSTAFF*)	−0.113	*BIG* *COVID*	1.218 **
	(−0.059)		(2.029)
(ln*MSTAFF*)(ln*FIXED*)	2.098	*YEAR*	0.249 ***
	(1.211)		(3.10)
		*YEAR* ^2^	0.019 ***
			(4.45)
Adjusted R-squared	0.822		
Degrees of freedom	148		
The null hypothesis (log-linear) ($\alpha_{il}=\beta_{11}=\delta_{11}=\epsilon_{11}=\gamma_{i1}=\varepsilon_{i1}=\mu_{i1}=\theta_{11}=\rho_{11}=\sigma_{11}=0$)			
*F*-statistic value	2.75		
Significance level	0.000		

Note: The significance levels are denoted by *, **, and *** representing the 10%, 5%, and 1% levels of significance, respectively; Please refer to Table 1 for the definitions of all variables used in this study.

**Table 5:** Average partial effects of variables.

APE	Value	Significance Test
*APE_MSTAFF*	−0.936	$H_{0}:\alpha_{1}=\alpha_{11}=\alpha_{12}=\alpha_{13}=\gamma_{11}=\varepsilon_{11}=\mu_{11}=0$
		*F*-stat. = 2.27
		Sign. level = 0.03
*APE_RSTAFF*	0.069	$H_{0}:\alpha_{2}=\alpha_{22}=\alpha_{12}=\alpha_{23}=\gamma_{21}=\varepsilon_{21}=\mu_{21}=0$
		*F*-stat. = 4.92
		Sign. level = 0.00
*APE_OSTAFF*	0.674	$H_{0}:\alpha_{3}=\alpha_{33}=\alpha_{13}=\alpha_{23}=\gamma_{31}=\varepsilon_{31}=\mu_{31}=0$
		*F*-stat. = 4.39
		Sign. level = 0.00
*APE_FIXED*	0.258	$H_{0}:\beta_{1}=\beta_{11}=\gamma_{11}=\gamma_{21}=\gamma_{31}=\theta_{11}=\rho_{11}=0$
		*F*-stat. = 4.38
		Sign. level = 0.00
*APE_DEVELOP*	0.044	$H_{0}:\delta_{1}=\delta_{11}=\varepsilon_{11}=\varepsilon_{21}=\varepsilon_{31}=\theta_{11}=\sigma_{11}=0$
		*F*-stat.=1.94
		Sign. level=0.07
*APE_BIOLOGY*	0.070	$H_{0}:\epsilon_{1}=\epsilon_{11}=\mu_{11}=\mu_{21}=\mu_{31}=\rho_{11}=\sigma_{11}=0$
		*F*-stat.=2.43
		Sign. level=0.02
*APE_BIG*		$H_{0}:\varphi_{1}=\varphi_{3}=0$
When *COVID* = 0	1.657	*F*-stat.=2.14
When *COVID* = 1	2.222	Sign. level=0.12
*APE_COVID*		$H_{0}:\varphi_{2}=\varphi_{3}=0$
When *BIG* = 0	0.628	*F*-stat.=3.58
When *BIG* = 1	1.464	Sign. level = 0.03
		$H_{0}:\varphi_{2}=\varphi_{3}=0$
*APE_YEAR*	0.211	*F*-stat. = 4.89
		Sign. level = 0.00

## Data Availability

The original contributions presented in the study are included in the article, further inquiries can be directed to the corresponding author.
